# Dynamic behavior of nematic liquid crystal mixtures with quantum dots in electric fields

**DOI:** 10.3762/bjnano.9.39

**Published:** 2018-02-01

**Authors:** Emil Petrescu, Cristina Cirtoaje, Octavian Danila

**Affiliations:** 1 University Politehnica of Bucharest, Department of Physics, Splaiul Independenţei 313, 060042, Bucharest, Romania

**Keywords:** Fréedericksz transition, nematic liquid crystals, quantum dots

## Abstract

The dynamic behavior of a mixture consisting of liquid crystalline 4-cyano-4’-pentylbiphenyl (5CB) and CdSe/ZnS quantum dots in electric fields was theoretically studied. The model was based on elastic continuum theory considering the interaction of the nematic molecules with the surrounding molecules, with the quantum dots and with the electric field. Experimental data obtained by dynamic measurements on a sample containing 0.89% (mass fraction) of CdSe/ZnS quantum dots revealed a decrease of the relaxation time compared to pure 5CB.

## Introduction

The expansion of liquid crystal (LC)-based devices in common life domains as well as science and engineering, requires improved technologies and new materials. Materials science provides great opportunities to these technologies by synthesizing new nanoparticles that can be mixed with liquid crystals: carbon nanotubes [1–3], graphene, magnetic nanoparticles [4–5], gold nanoparticles [6], quantum dots (QDs) [7–12] or other nanomaterials [13–17] that can be effectively used in electro-optical devices.

When added to liquid crystals, quantum dots may seriously influence their behavior under an electric field due to the anchoring forces acting on the quantum dot surface. Dynamic experiments performed in alternating electric fields proved that by adding a small amount of CdSe/ZnS quantum dots in thermotropic nematic liquid crystal with positive dielectric anisotropy, we obtain a decrease of the relaxation time.

When an external electric field higher than the Fréedericksz transition threshold [18–19] is applied transversely to a planar liquid crystal cell, the molecules change their orientation tending to align their director in parallel to the field. This reorientation is illustrated by an intensity variation of a laser beam crossing through the sample. The time period after which a constant intensity is obtained, which means that an equilibrium state is reached, is characterized by the relaxation time and can be experimentally evaluated from plotting the intensity as a function of the time. A theoretical analysis of the LC + QDs system based on elastic continuum theory allowed us to express the time evolution of the molecular distortion angle inside the cell and to calculate the relaxation time. Theoretical values and experimental data were in good agreement showing that the model offers an important tool for engineers to precisely control LC-based device parameters.

## Theoretical Background

When a nematic liquid crystal with positive dielectric anisotropy is exposed to an electric field, and if the intensity of the field is higher than the Fréedericksz transition threshold, the molecular director changes its orientation, trying to align in parallel with the field direction. During this reorientation process, the refractive index of the sample also changes:

[1]
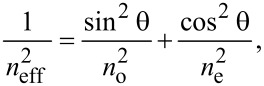


where *n*_o_ and *n*_e_ are, respectively, the ordinary and extraordinary refractive indexes and θ is the angle between the direction of the light and *n*_e_. Between the extraordinary and ordinary rays, there is a path difference which can be calculated as a function of the cell thickness, *L*:

[2]
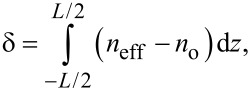


with the corresponding phase difference Δφ = (2πδ)/λ.

For a planar cell configuration we have:

[3]
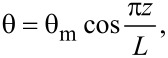


where θ_m_ is the maximum deviation angle (in the middle in the cell). If small deviation angles are considered, the phase difference becomes:

[4]
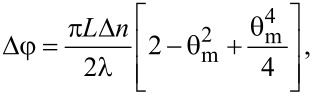


and the intensity of the laser beam crossing through the sample is:

[5]
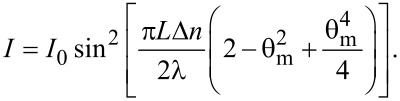


The equation of the maximum deviation angle as a function of time is obtained from the total free energy of the system by applying the Euler–Lagrange equation. Elastic continuum theory states that a liquid crystal is acting like a continuous fluid and the interaction forces between its molecules are elastic. Taking into account a strong anchoring on the glass support, the free energy density of such a system with added quantum dots is:

[6]
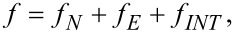


where *f_N_* is the liquid crystal free energy density, *f_E_* is a term combining the electric field action on the liquid crystal molecules in the presence of quantum dots and *f_INT_* reflects the interaction between quantum dots and liquid crystal molecules.

**Figure 1:**
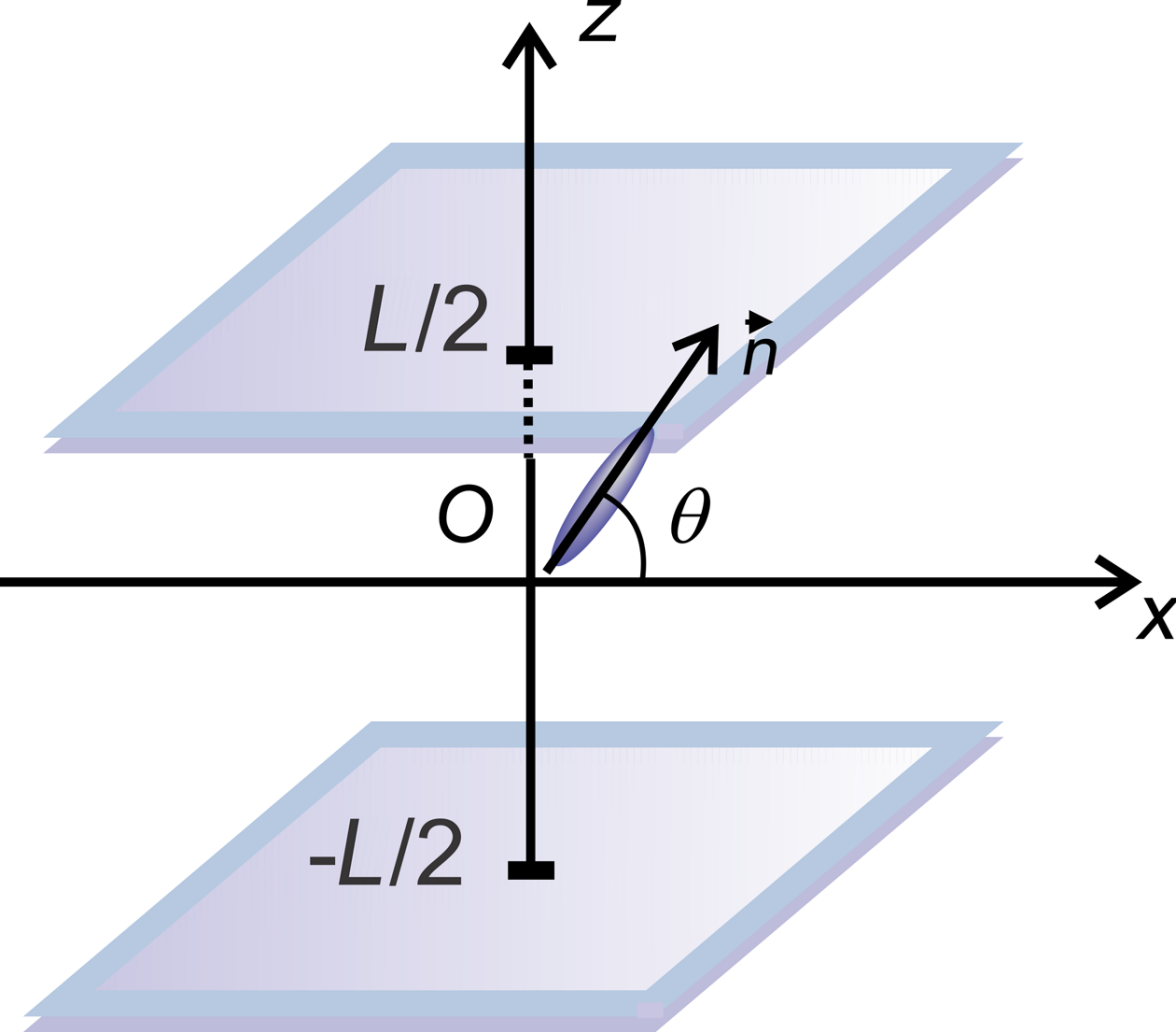
Molecular director orientation of a liquid crystal with a positive anisotropy in a planar cell exposed to an electric field.

For a planar aligned cell (Figure 1) with thickness *L*, and when the voltage is just above the Fréedericksz transition threshold, the deviation angle is very small and the free energy caused by the elastic forces is:

[7]
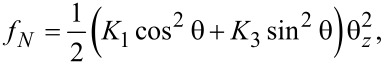


where *K*_1_ and *K*_3_ are the splay and bent elastic constants and θ*_z_* = ∂θ/∂*z*.

Due to the presence of the QDs, the electric properties of the mixture change. Hence, instead of perpendicular dielectric permittivity, 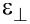, and electric anisotropy, ε*_a_*, we use the effective perpendicular permittivity, 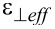, and effective anisotropy, ε*_aeff_*.

[8]
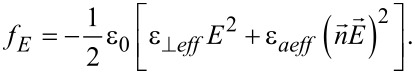


If a small spherical cavity is considered inside the liquid crystal, a polarisation field on the hole surface appears. According to classical theory developed by Landau and Lifshitz [20], the polarization field around a spherical cavity inside a dielectric medium is proportional to the applied field, so:

[9]
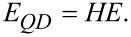


Inside an anisotropic dielectric (the liquid crystal) the spherical cavity changes into an ellipsoid with the semi-axes 
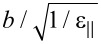
 and 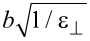, where *b* is the cavity radius, 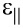 is the parallel permittivity, 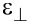 is the perpendicular permittivity. In this case *H* becomes:

[10]
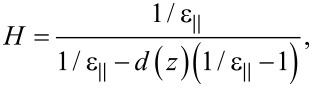


where *d*(*z*) the depolarization coefficient, which for an ellipsoid with almost spherical shape (horizontal axis equal *b* = *R* and vertical axis *a* = *R* + 0.0005*R*) is


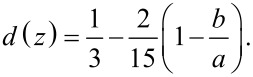


If a quantum dot is placed inside this cavity, another polarization field will occur but because of its semiconductor nature, this field is much smaller than the one produced at the surface of the dielectric and might be neglected. Thus, the effective field acting on the nematic molecules is smaller than the applied field *E* by *E_QD_*, i.e., *E*′ = *E* − *HE*.

The free energy density may also be written as:

[11]
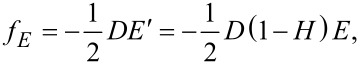


where *D* is the electric induction. Thus the free energy is:

[12]
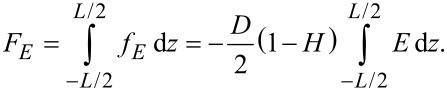


**Figure 2:**
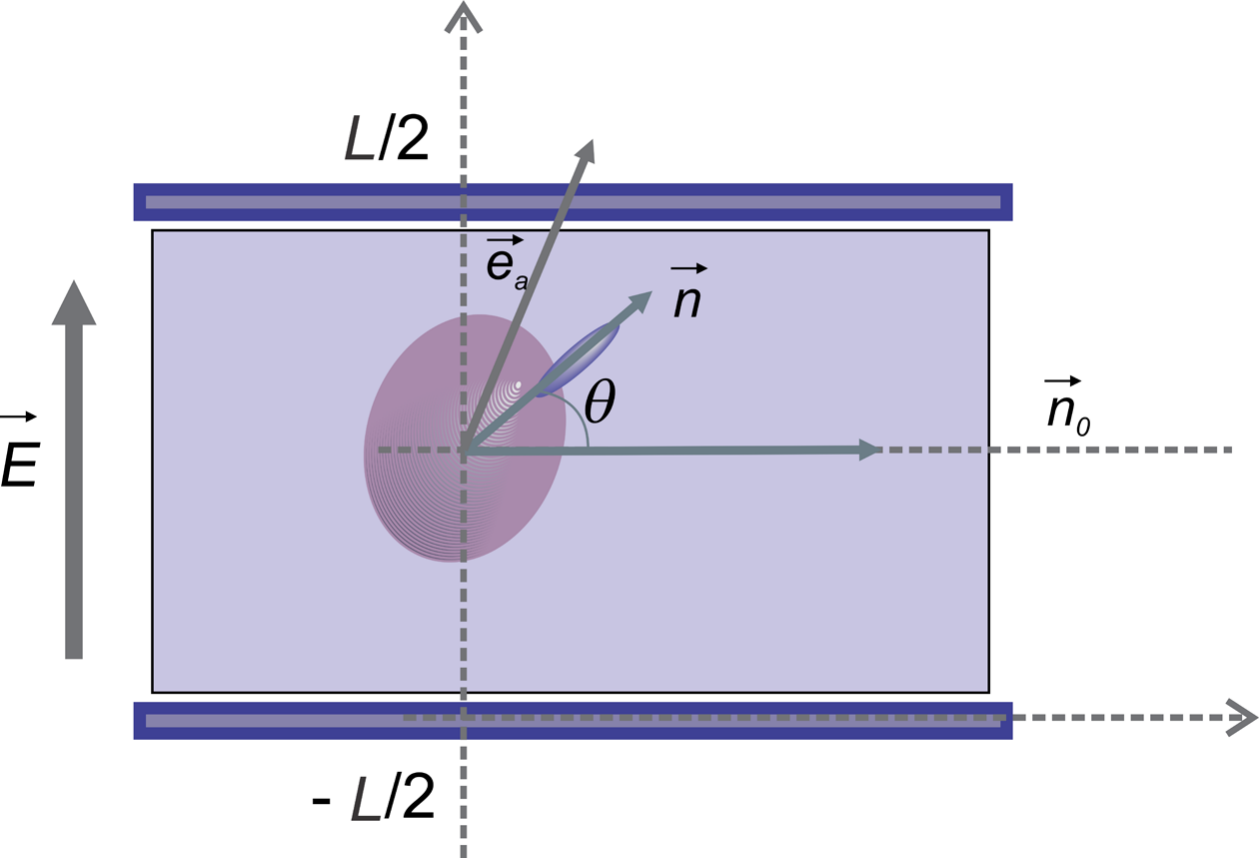
Molecular director and quantum dot orientation in a planar LC cell subjected to electric field.

By taking into account that the applied voltage on the sample is


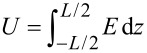


and considering small angle approximation for the configuration presented in Figure 2 we get:

[13]
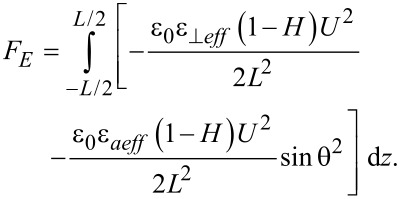


Then the free energy density is:

[14]
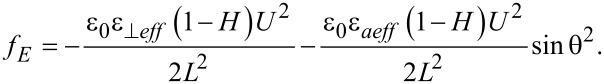


The first term does not depend on the molecular orientation so we may neglect it because it disappears when applying the Euler–Lagrange equation. Thus, we may write:

[15]
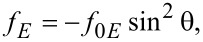


where

[16]
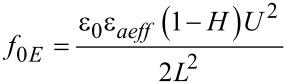


and

[17]
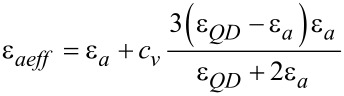


is the effective dielectric anisotropy of the liquid crystal containing quantum dots as a function of the dielectric anisotropy of the LC, (ε*_a_*), of the dielectric constant of the QDs (ε*_QD_*) and the volumetric fraction of the quantum dots (*c_v_*).

**Figure 3:**
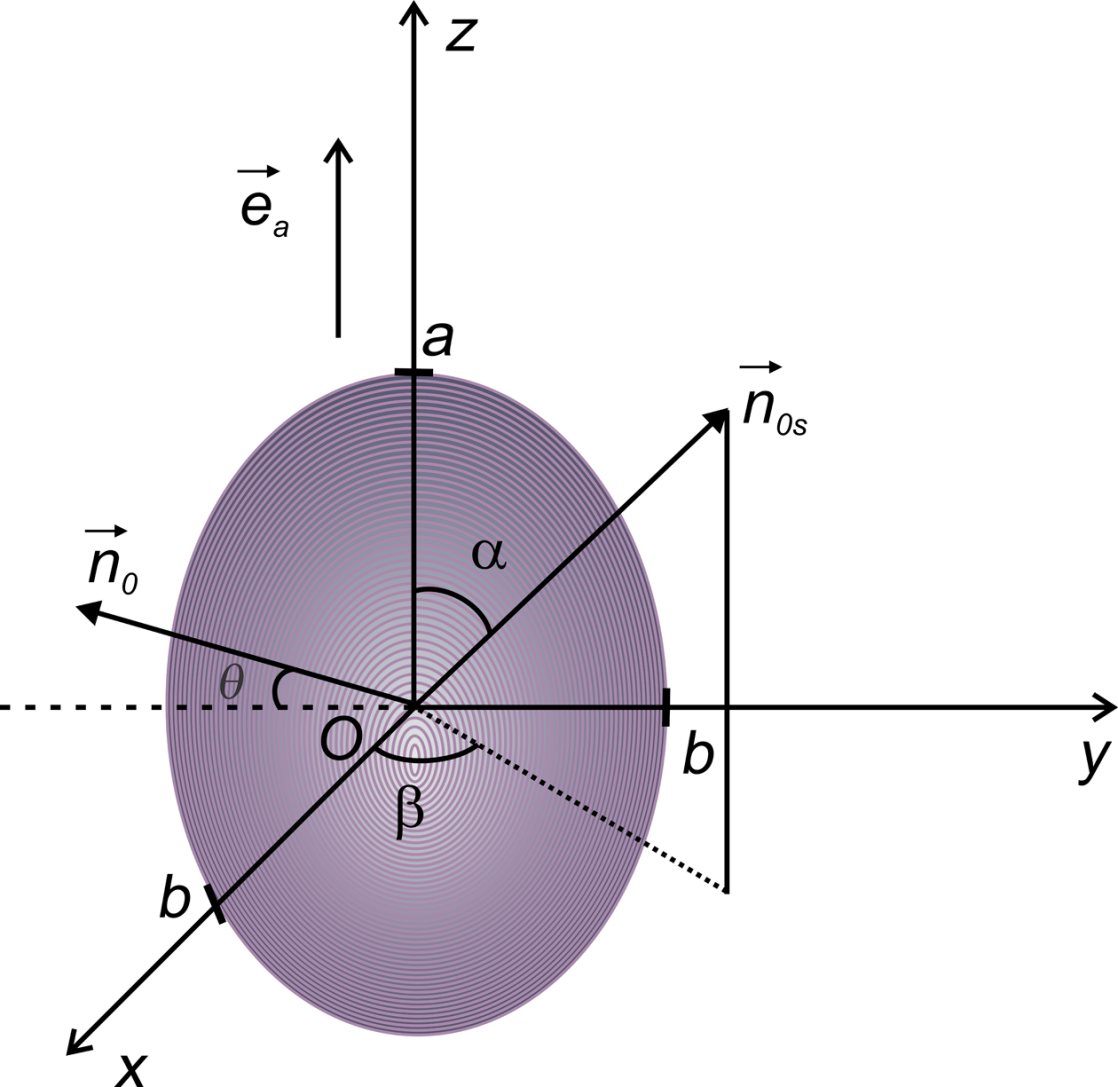
Nematic molecular orientation around an ellipsoidal QD.

The intraction free energy between nematic molecules and a quantum dot can be calculated from the Rapini formula [21]. Considering an elliptical quantum dot with one of the axes just a little bit longer than the others and the configuration given in Figure 3, as well as homeotropic anchoring of liquid crystal molecules on the QD surface [12], the interaction energy for a particle can be obtained using a similar procedure as presented by Burylov [22]:

[18]
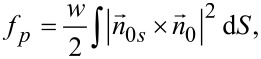


where *w* is the anchoring energy on the surface of the nanoparticle, 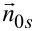 is the nematic director on the QD surface and 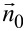 is the direction of molecular director far away from the QD surface.

According to the manufacturers, the quantum dots are spherical particle with the radius *R* = 3 nm (6 nm in diameter). Considering their small anisotropy we approximate the sphere with an almost spherical ellipsoid having a short axis (*b* = *R*) and a long axis close to *b*. Thus, we obtain:

[19]
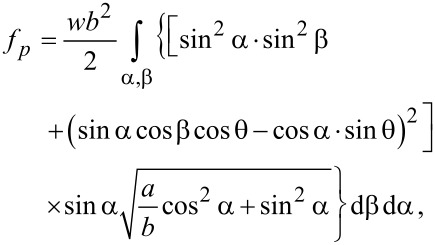


This equation can be solved and written in a simplified form as:

[20]
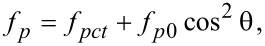


where *f_pct_* is a constant depending on the anchoring energy and the particle geometry and disappears when applying the Euler–Lagrange equations. Thus, it may be neglected and *f_p0_* = 11.52*wb*^2^π . Considering the volumetric concentration of quantum dots, we obtain:

[21]
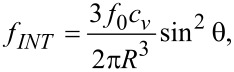


where *f*_0_ = (11.52/2)*w*π*R*^2^ is a constant depending on the geometrical parameters of the system and the LC + QD interaction energy calculated for the ellipsoid QD presented in Figure 3. When a dynamic study of the mixture is made, the total free energy density must also take into account the dissipative time-dependent term, and the total free energy becomes:

[22]
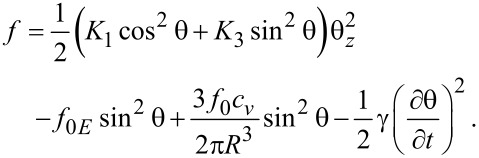


By applying the Euler–Lagrange equation and assuming that θ is small we obtain:

[23]
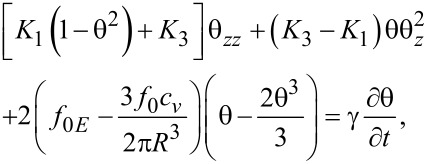


where θ*_zz_* = ∂^2^θ/∂*z*^2^. Considering the deviation angle from Equation 3, we obtain the dependency of the deviation angle on the relaxation time:

[24]
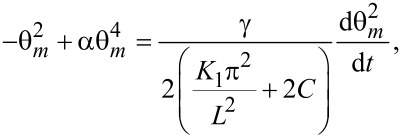


where


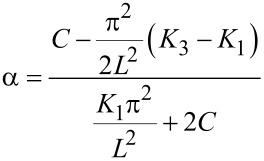


and


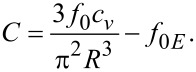


Solving this equation leads to

[25]
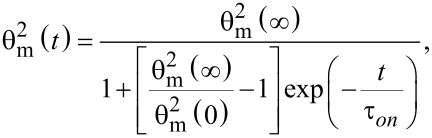


where θ_m_(∞) and θ_m_(0) are the limit values of the deviation angle in the center of the cell and τ*_on_* is:

[26]
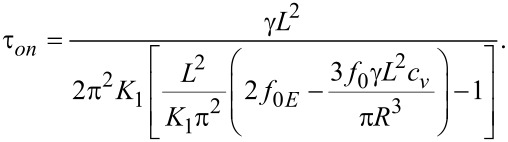


When the voltage is switched off, we follow the same procedure after replacing *f_0E_* = 0 in Equation 22. Thus, we obtain:

[27]
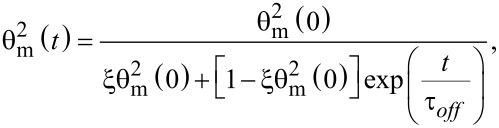


where θ_m_(0) is the maximum deviation angle at *t* = 0, ξ is a constant depending on LC properties and τ*_off_* is:

[28]
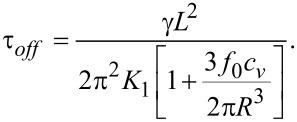


## Experimental

**Figure 4:**
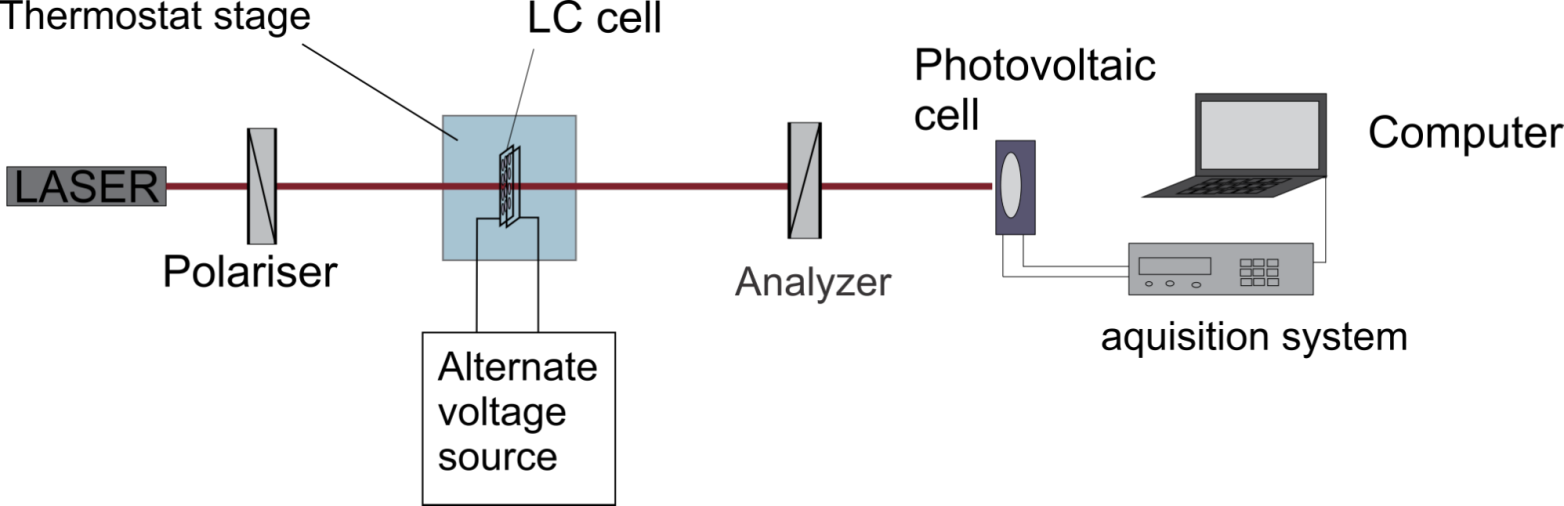
Experimental setup for the dynamic study of the LC + QDs mixture.

For the sample preparation we used CdSe/ZnS quantum dots in toluene with an average size of 6 nm and a fluorescence wavelength of 630 nm from Aldrich. The QD suspension was left in a bottle in clean vacuum environment until the toluene was completely evaporated. Then, 4-cyano-4’-pentylbiphenyl (5CB) was added and sonicated for three days, several hours each day until a homogenous pink mixture was obtained. The homogeneity was checked by using polarization microscopy. The mass concentration was 0.89%, which led to a volumetric fraction of 1.16% (the QD density is 0.87 g/cm^3^ and the density of 5CB is 1.02 g/cm^3^). The mixture was used to fill a 15 μm thick planar aligned cell from Instek, which was mounted in a holder placed in a Mettler Toledo hot stage. A reference sample was made using pure 5CB. The terminals of the holder were connected to a power source that produced a square wave signal with a frequency of 10 kHz. For voltages higher than the Fréedericksz transition threshold, the nematic director changes its orientation trying to align with the field. A He–Ne laser beam crossed the sample through the windows of the holder and the emergent signal was recorded with a Thor Lab photovoltaic cell. Two crossed polarizers at 45° were placed on both sides of the sample to obtain equal intensities for ordinary and extraordinary rays. An acquisition system connected to a computer allowed data recording every 20 ms. A schematic representation of the experimental setup is presented in Figure 4.

## Results and Discussion

**Figure 5:**
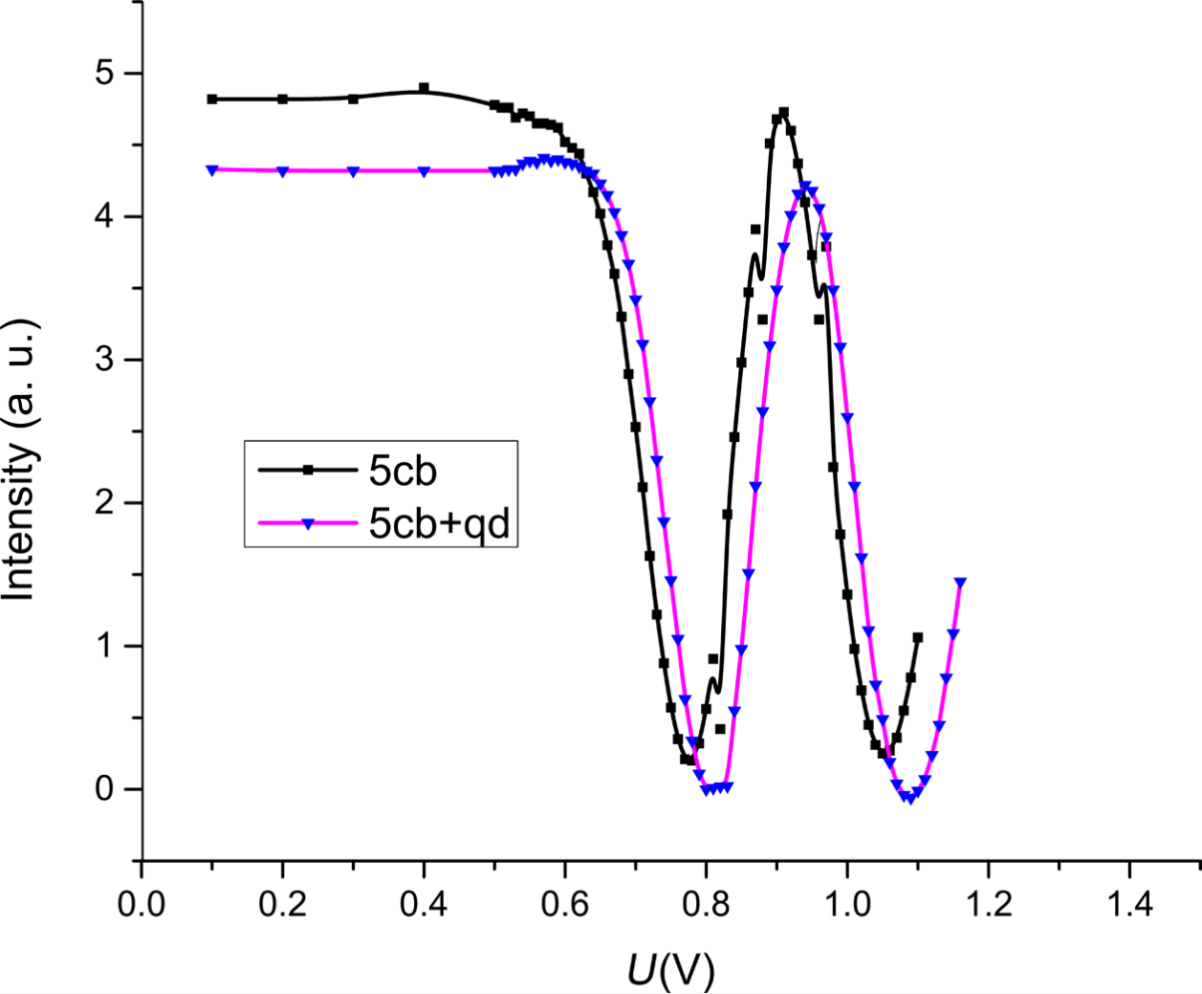
Fréedericksz transition for 5CB and 5CB + QD. Lines are guide to the eyes.

Fréedericksz transition threshold and relaxation time were determined for both samples at a constant temperature of 28 °C. The Fréedericksz transition threshold was determined by slowly increasing the applied voltage and while recording the laser intensity. Preliminary measurements showed no changes of the laser intensity below 0.5 V. Hence, the voltage steps were 0.1 V below this value and 0.01 V above it. The data were represented in an intensity versus voltage plot (Figure 5). Above the threshold value, the intensity of the emergent laser beam exhibits a series of maxima and minima.

For the dynamic measurements the value of the first intensity maximum was chosen. Thus, the applied voltage was without any doubt above the Fréedericksz transition and the deviation angle was small as considered in the theoretical model. The intensity versus time plot was recorded for each sample and the function in Equation 5 was used to fit the data. A power switch was connected to the acquisition system so that when the field was switched on the acquisition started at the same time to measure the intensity when the field is switched on. A similar procedure was used when the power source was disconnected and at the same time the acquisition started to measure intensity versus time plot when the field was switched off.

**Figure 6:**
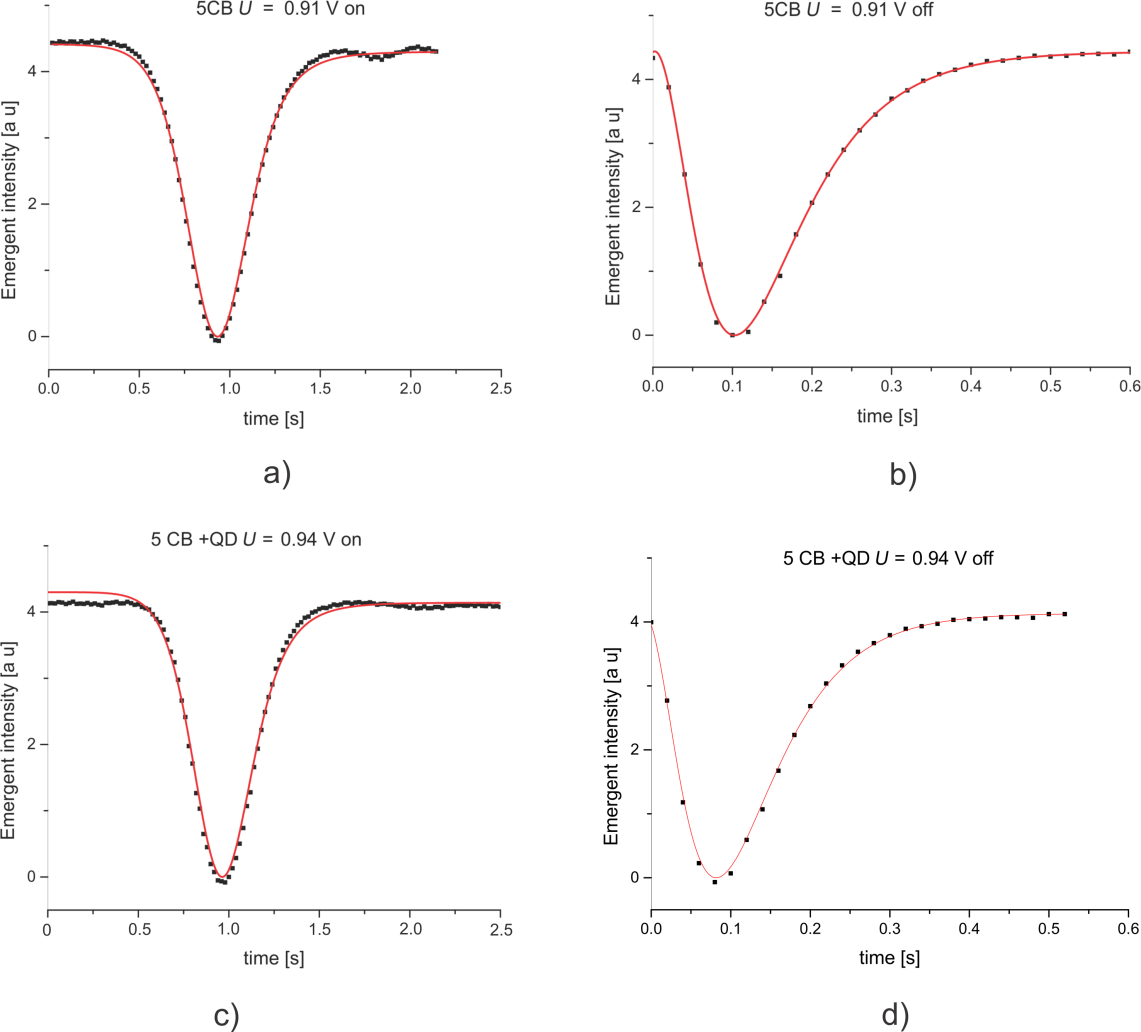
Dynamic measurement of emergent light intensity: a) for LC sample when the field is on; b)for LC sample when the field is off; c) for LC + QDs sample when the field is on; for LC + QDs sample when the field is off. The dots represent experimental values and the red lines are theoretical representations.

In Figure 6 we presented the light intensity versus time plot for the pure LC (Figure 6a,b) and for the mixture of LC with QDs (Figure 6c,d). Experimental data were fitted using Equation 5 with the time dependency of *theta*_m_ given by Equation 25 and Equation 27, where *L* = 15 μm, *K*_1_ = 6.2 × 10^−12^ N, *R* = 3 nm and *c_v_* = 1.16% (volumetric fraction). Therefore, we obtain for the sample containing QDs, the experimental relaxation time τ*_on−exp_* = 0.167 s when the field is switched on and τ*_off−exp_* = 0.139 s when the field is switched off.

By fitting the data of the 5CB sample, we obtained a relaxation time τ*_on−exp0_* = 0.172 s when the field is switched on and τ*_off−exp0_* = 0.157 s when the field is switched off. The relaxation time decreases when quantum dots are added. Generally, this result was reported in similar researches [9–11] but only when the field was switched on. When the field was off the results in [9] showed an increase of the relaxation time for the sample containing quantum dots. The measurements were made on similar systems both of which emplyed CdSe/ZnS in 5CB but in our case a temperature of 28 °C yielded a lower viscosity and, comsequently, a lower relaxation time especially when the field is switched off. In [8] the authors demonstrate an increase of the elastic coefficient *K*_1_ with the QD concentration, which is in good agreement with Equation 26 and Equation 28 where the effective elastic constant is proportional with the volumetric fraction of the added quantum dots.

Basu and co-workers [7] proposed a different model of a quantum dot dispersion in a nematic liquid crystal. According to this model, dots are gathering together forming long chains around which liquid crystal molecules are homogeneously aligned. Such an alignment could not explain the decrease of the relaxation time when the field is switched on because the anchoring forces on quantum dots surface will slow down the molecular movement.

**Figure 7:**
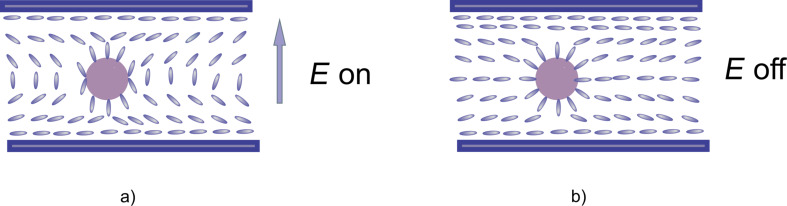
Schematic representation of the molecular orientation around a QD a) when the field is switched on b) when the field is switched off.

A possible explanation for this behavior might be given considering a homeotropic alignment of the LC molecules on the QD surface [12]. Thus, if the applied field is high enough to exceed the Fréedericksz transition threshold some of the molecules are already parallel to the field and only those placed on the horizontal sphere circumference are parallel to the cell plates, so the response time is shorter in the quantum dots sample (Figure 7a). When the field is switched off, all the molecules are distorted and their returning to the original position is “helped” by the anchoring forces on the QD surface and a slight decrease of the relaxation time is observed (Figure 7b).

## Conclusion

We observed an improvement in the turn-off time of the nematic liquid crystal 5CB containing CdSe/ZnS quantum dots of a nominal size of 6 nm diameter. The results are potentially useful in other cell geometries and with other materials. The theoretical model might be extended to other geometries and used to theoretically optimize the performance of QD–liquid crystal composites. The calculations presented in this paper are based on particular experimental results obtained at 28 °C, for a relatively high concentration of quantum dots. Further measurements are to be done in the future, over a wide temperature range and under consideration of other parameters, to improve this model and obtain an accurate theoretical description of these systems.

## References

[1] Cirtoaje C, Petrescu E (2016). Physica E.

[2] Samoilov A N, Minenko S S, Fedoryako A P, Lisetski L N, Lebovka N I, Soskin M S (2014). Funct Mater.

[3] Bale S, Liyana-Arachchi T P, Hung F R (2016). Mol Simul.

[4] Cirtoaje C, Petrescu E, Stan C, Creanga D (2016). Physica E.

[5] Mănăilă-Maximean D, Cîrtoaje C, Dănilă O, Donescu D (2017). J Magn Magn Mater.

[6] Lewandowski W, Fruhnert M, Mieczkowski J, Rockstuhl C, Górecka E (2015). Nat Commun.

[7] Basu R, Iannacchione G S (2009). Phys Rev E.

[8] Vakulin D A, Frenkel D A, Gavrish E O, Konshina E A (2015). Mol Cryst Liq Cryst.

[9] Konshina E A, Galin I F, Shcherbinin D P, Gavrish E O (2014). Liq Cryst.

[10] Gavrish E O, Galin I F, Konshina E A, Vakulin D A (2015). Mol Cryst Liq Cryst.

[11] Galin I F, Konshina E A (2015). Mol Cryst Liq Cryst.

[12] Prodanov M F, Pogorelova N V, Kryshtal A P, Klymchenko A S, Mely Y, Semynozhenko V P, Krivoshey A I, Reznikov Y A, Yarmolenko S N, Goodby J W (2013). Langmuir.

[13] Cîrtoaje C, Petrescu E, Stoian V (2015). Physica E.

[14] Motoc C, Cirtoaje C, Stoica A, Stoian V, Albu A M (2013). UPB Sci Bull, Ser A.

[15] Zakhlevnykh AN, Lubnin M S, Petrov D A (2008). J Magn Magn Mater.

[16] Ganea C, Manaila-Maximean D (2011). UPB Sci Bull, Ser A.

[17] Manaila-Maximean D, Rosu C, Yamamoto T, Yokoyama H (2004). Mol Cryst Liq Cryst.

[18] Fréedericksz V, Repiewa A (1927). Z Phys.

[19] Fréedericksz V, Zolina V (1933). Trans Faraday Soc.

[20] Landau L D, Lifshitz E M (1960). Electrodynamics of Continuous Media.

[21] Rapini A, Papoular M (1969). J Phys Colloques.

[22] Burylov S V, Raikher Yu L (1993). J Magn Magn Mater.

